# Milk-derived extracellular vesicles enable gut-to-tumor oral delivery of tumor-activated doxorubicin prodrugs

**DOI:** 10.7150/thno.97269

**Published:** 2024-08-26

**Authors:** Hochung Jang, Jiwoong Choi, Daeho Park, Geonhee Han, Eun Hye Kim, Kwangmeyung Kim, Sun Hwa Kim, Man Kyu Shim, Yoosoo Yang

**Affiliations:** 1 Medicinal Materials Research Center, Biomedical Research Division, Korea Institute of Science and Technology (KIST), Seoul, 02792, Republic of Korea.; 2 Division of Bio-Medical Science and Technology, KIST School, Korea University of Science and Technology, Seoul 02792, Republic of Korea.; 3 Department of Life Sciences, Korea University, Seoul, 02841, Republic of Korea.; 4 College of Pharmacy, Graduate School of Pharmaceutical Sciences, Ewha Womans University, Seoul 03760, Republic of Korea.; 5 KU-KIST Graduate School of Converging Science and Technology, Korea University, Seoul, 02841, Republic of Korea.

**Keywords:** cancer therapy, extracellular vesicles, oral drug delivery, prodrug, chemotherapy

## Abstract

**Rationale:** Oral chemotherapy has been emerging as a hopeful therapeutic regimen for the treatment of various cancers because of its high safety and convenience, lower costs, and high patient compliance. Despite the current advancements in nanoparticle-mediated drug delivery, numerous anticancer drugs susceptible to the hostile gastrointestinal (GI) environment exhibit poor permeability across the intestinal epithelium, rendering them ineffective in providing therapeutic benefits. In this paper, we focus on harnessing milk-derived extracellular vesicles (mEVs) for gut-to-tumor oral drug delivery by leveraging their high bioavailability.

**Methods:** The tumor-activated prodrug (a cathepsin B-specific cleavable FRRG peptide and doxorubicin, FDX) is used as a model drug and is complexed with mEVs, resulting in FDX@mEVs. To verify stability in the GI tract, prolonged intestinal retention, and enhanced trans-epithelial transport via neonatal Fc receptor (FcRn)-mediated transcytosis, intestinal transport evaluation is conducted using *in vitro* intestinal barrier model and mouse model.

**Results**: FDX@mEVs form a stable nanostructure with an average diameter of 131.1 ± 70.5 nm and complexation processes do not affect the inherent properties of FDX. Orally administered FDX@mEVs show significantly improved bioavailability compared to uncomplexed FDX via FcRn-mediated transcytosis of mEVs resulting in increased tumor accumulation of FDX in tumor-bearing mouse model.

**Conclusions:** After oral administration of FDX@mEVs, it is observed that remarkable antitumor efficacy in colon tumor-bearing mice without adverse effects, such as body weight loss, liver/kidney dysfunction, and cardiotoxicity.

## Introduction

Oral delivery is favored for its convenience, non-invasiveness, and cost-effectiveness 1, 2. In addition to improving patient compliance, the oral route also holds significance from a physiological perspective 3. The gastrointestinal (GI) tract, with its expansive surface area (300-400 m^2^), facilitates drug absorption for systemic exposure and target-specific drug delivery 4. Moreover, the intestinal epithelium features highly expressed receptors, such as the neonatal FC receptor (FcRn), promoting significant drug absorption through transcytosis. This process involves apical endocytosis, intracellular trafficking, and basolateral exocytosis in a sequential mechanism 5, 6. However, achieving sufficient drug concentration in target regions via oral administration is challenging due to various biological barriers in the GI tract involving extensive enzymatic degradation, poor penetration of drugs across the GI tissue barrier, metabolic processes during systemic circulation, and rapid excretion 7. Indeed, it has been reported that approximately 70% of novel drug candidates face discontinuation during preclinical studies due to low oral bioavailability 8. Hence, there is a pressing need for oral delivery systems to enhance drug bioavailability and improve therapeutic outcomes.

One of the most promising strategies to overcome these challenges in oral delivery is the employment of nanoparticulate systems 9, 10. Among various nanoparticles, extracellular vesicles have gained prominence as effective drug carriers due to their capacity to encapsulate both hydrophilic/hydrophobic molecules and to pass biological barriers via membrane-associated proteins 11, 12. Furthermore, the CD47 proteins expressed on the surface of extracellular vesicles (EVs) emit a “do not eat me” signal, preventing *in vivo* phagocytosis of EVs. This phenomenon allows EVs carrying drugs to evade premature elimination by the mononuclear phagocyte system, enabling effective drug delivery to the target tissues with minimal dosage 13.

EVs, derived from various sources such as cells, foods, or mammalian bodily fluids, exhibit significant differences in composition and natural function depending on their origins 14. Milk, in particular, emerges as a superior and scalable alternative, outperforming conventional EVs sources in terms of both bulk production and cost-effectiveness 15, 16. Milk-derived extracellular vesicles (mEVs), boasting yields over 20 times higher than cell-derived counterparts 17, exhibit considerable potential for oral drug delivery, enduring harsh conditions of the GI tract, including its highly acidic pH and diverse digestive enzymes 18, 19.

**Scheme 1:**
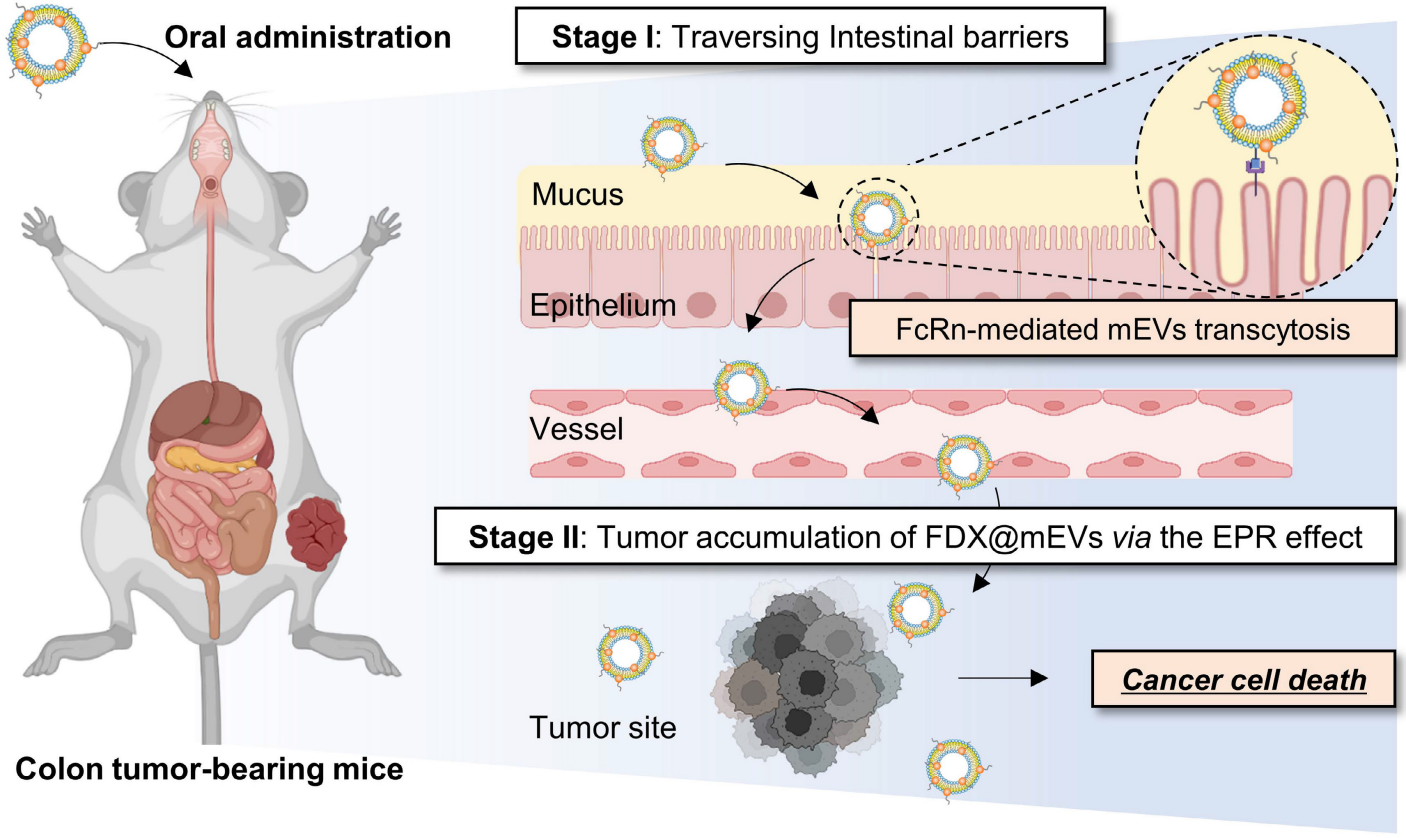
** Schematic showing mEVs-mediated FDX oral delivery for cancer therapy.** The effective anticancer efficacy of orally administered FDX, facilitated by mEVs, is primarily attributed to a two-stage process. Initially, the interaction between the IgG present on the mEVs and the FcRn in the intestinal epithelium induces transcytosis of FDX@mEVs, thereby enhancing the limited bioavailability of FDX and elevating its systemic exposure. Subsequently, the enhanced bioavailability of FDX, mediated by mEVs, contributes to its accumulation in tumor sites. This phenomenon results in increased antitumor effects of FDX@mEVs.

Herein, we reveal that mEVs exhibit a unique mechanism to facilitate systemic exposure through interaction with the FcRn in the intestines following oral administration, enabling effective gut-to-tumor oral drug delivery (**Scheme 1**). Possessing remarkable stability in the GI tract, FDX@mEVs, a complex of mEVs with tumor-activated doxorubicin prodrugs (FDX), exhibited prolonged intestinal retention. This led to their entry into the bloodstream through FcRn-mediated transcytosis, ultimately resulting in extensive accumulation of FDX@mEVs within tumors; once inside the tumor, the prodrugs comprising of an FRRG (Phe-Arg-Arg-Gly) peptide and doxorubicin undergo an enzymatic cleavage by cathepsin B, which releases free DOX specifically in tumors and induces apoptotic cell death without off-target effects. Taken together, our findings showcase a versatile and reliable oral delivery strategy using mEVs for effective and safe cancer therapy with enhanced patient compliance.

## Results and Discussion

### Preparation and physicochemical characterization of FDX@mEVs

**Figure 1:**
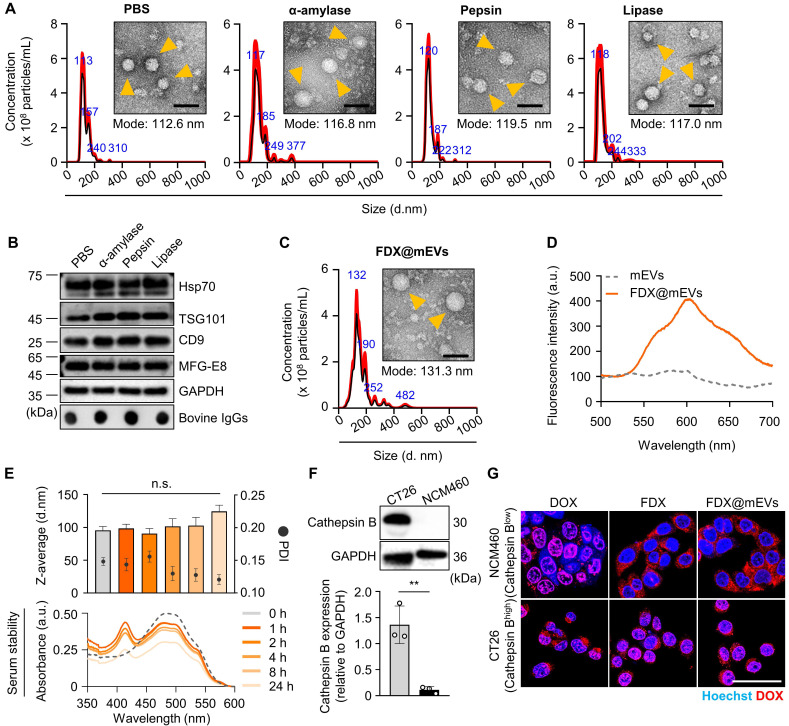
**Preparation and physicochemical characterization of FDX@mEVs. (A)** Hydrodynamic size distribution, TEM images of mEVs after incubation in digestive enzymes. Orange arrowheads indicate mEVs. Scale bar: 100 nm.** (B)** Immunoblotting analysis for detecting EV markers (TSG101, Hsp70, CD9, and MFG-E8), bovine IgG. **(C)** TEM image and hydrodynamic size distribution of FDX@mEVs. Orange arrowheads indicate FDX@mEVs. Scale bar: 100 nm. **(D)** Fluorescence spectra of mEVs and FDX@mEVs in the same fraction. **(E)** Particle size, PDI (upper), and UV-vis spectra (below) of FDX@mEVs complex after serum incubation at various time points (0, 1, 2, 4, 8, and 24 h). **(F)** Western blotting analysis for assessing cathepsin B expression levels in CT26 and NCM460 cells. **(G)** Representative fluorescence images showing the internalization of FDX in cell nuclei. Scale bar: 100 μm. All data presented mean ± SD.

As biomimetic nanovesicles for oral drug delivery from the gut to tumors, FDX@mEVs were prepared through a three-step protocol involving EV isolation, prodrug synthesis, and their complexation. Initially, we investigated the stability of mEVs obtained from pasteurized low-fat milk in the presence of various digestive enzymes encountered in the GI tract following oral administration. The nanoparticle tracking analysis (NTA) results showed that the mEVs maintained a constant mode size distribution similar to their intrinsic characteristics in PBS (112.6 ± 30.3 nm) after 30 min of incubation in α-amylase (116.8 ± 47.2 nm), pepsin (119.5 ± 19.3 nm), and lipase (117.0 ± 37.3 nm; **Figure 1A**). As a result of checking the EV proteins listed in Minimal information for studies of extracellular vesicles 2018 (MISEV2018) 20, there were also no significant changes in the expressions of EV markers, tumor susceptibility gene 101 (TSG101), CD9, and heat shock protein 70 (Hsp70) observed after 30 min of incubation in enzymes compared to those in PBS (**Figure 1B**). MFG-E8, which was used to verify that the exosome extraction source is milk, was also confirmed to be well maintained after enzymatic incubation, similar to the other previously mentioned EV markers. Furthermore, we confirmed that the IgG on the EV surface, capable of facilitating transcytosis from the GI tract into the bloodstream upon binding with FcRn, remained intact when incubated with such digestive enzymes 13, 21. The remarkable stability of mEVs in these digestive enzymes can potentially lead to significant accumulation within the GI tract with no structural decomposition.

Next, the tumor-activated doxorubicin prodrug FDX was synthesized by a simple one-step chemical conjugation of the cathepsin B-specific cleavable FRRG peptide with doxorubicin (DOX; **Figure S1A**). We previously found that FDX spontaneously self-assembles into nanoparticles with an average size of 220 nm via intermolecular π-π stacking and hydrophobic interactions without any additional carrier materials 22, 23. In this study, FDX was complexed with mEVs via passive incubation in PBS containing 10% dimethyl sulfoxide (DMSO), in which the prodrug exists in a soluble form (**Figure S1B**). The incubation in a 10% DMSO condition (*v/v*) did not alter the size distribution and concentration of mEVs (**Figure S2**). The purified FDX@mEVs exhibited a spherical morphology with a mode size of 131.3 ± 70.5 nm, the typical size distribution of EVs (**Figure 1C**). Additionally, a stability test using an *ex vivo* digestive system, which simulates the continuous action of digestive enzymes, confirmed that both mEVs and FDX@mEVs remained stable after digestion (**Figure S3**). The fluorescence spectra of FDX@mEVs revealed a strong DOX fluorescence compared to uncomplexed mEVs, indicating successful FDX complexation with mEVs (**Figure 1D**). To investigate the complexation model of FDX@mEVs, we performed single-particle analysis using super-resolution microscopy (SRM). SRM imaging results showed that FDX (green) exhibited a high correlation coefficient (0.5227 to 0.9441) with more than half of the mEVs (red) area (**Figure S4**), confirming their complexation. This complexation model is likely due to the hydrophobic part of FDX, Dox, integrating into the mEVs membrane. The drug complexation efficiency and capacity were determined to be 59.78 and 34.41%, respectively (see formula in the “Methods” section). FDX@mEVs displayed stability in PBS for 24 h, with no significant changes in particle sizes (**Figure 1E**). UV-vis absorbance results demonstrated that no detectable levels of prodrug release were observed when incubating FDX@mEVs in mouse serum for 24 h (**Figure 1E**). This finding alleviates concerns about potential premature drug leakage during blood circulation.

We then investigated the selective release of free DOX in tumor cells by FDX@mEVs through the target enzyme-specific cleavage of prodrugs. NCM460 human colon mucosal epithelial cells and CT26 murine colon cancer cells, expressing relatively low and high levels of cathepsin B, were treated with free DOX, FDX, and FDX@mEVs at an equivalent dose of 1 μM DOX (**Figure 1F**). After 6 h of treatments, FDX or FDX@mEVs exhibited DOX fluorescence mainly in the nuclei of CT26 cells, whereas NCM460 cells showed cytoplasmic signals (**Figure 1G**). In contrast, free DOX displayed similar intracellular behavior in both cell types regardless of cathepsin B expression levels, indicating potential cathepsin B-specific cytotoxicity induction by FDX@mEVs in tumor cells. To thoroughly investigate the cathepsin B-specific activation of FDX, we treated CT26 cells with the cathepsin B inhibitor CA-074, followed by treatment with FDX@mEVs. Confocal imaging results showed that in CA-074-treated CT26 cells, where cathepsin B activity was inhibited, FDX did not translocate into the nucleus (**Figure S5**). This specificity can reduce the potential risk of off-target toxicities in normal tissues with lower cathepsin B expression, where FDX@mEVs non-specifically localize.

### *In vivo* intestinal absorption of FDX@mEVs

**Figure 2:**
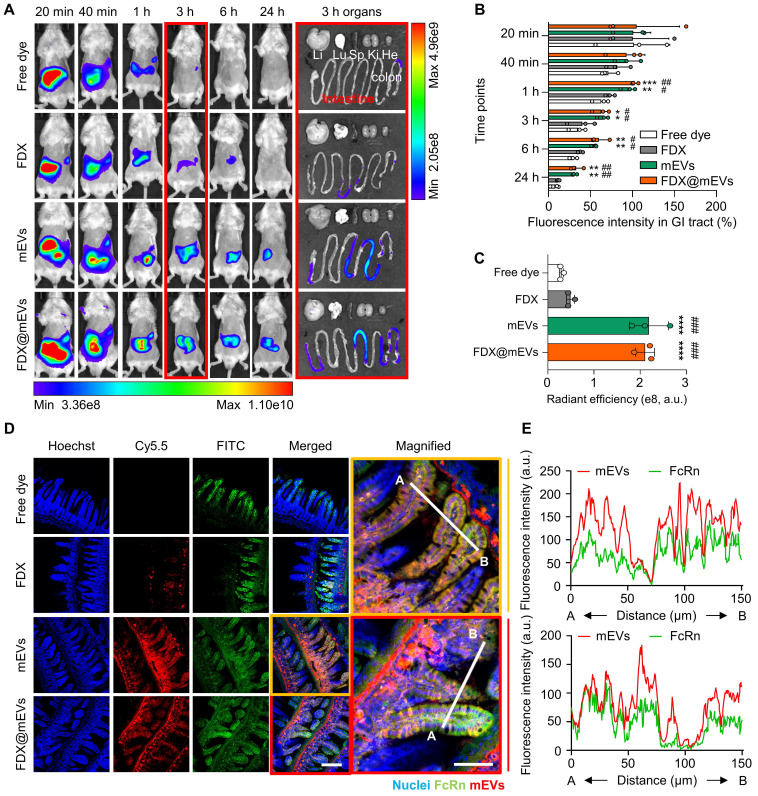
** Biodistribution of mEVs and histological analysis of the interaction between FcRn and mEVs in the intestinal region. (A)** *In vivo* whole-body NIRF images after oral administration of Free dye, FDX, mEVs, and FDX@mEVs at various time points (20, 40 min, 1, 3, 6, and 24 h) and *ex vivo* NIRF images of major organs (liver (Li), lungs (Lu), spleen (Sp), kidneys (Ki), and heart (He) including the GI tract. **(B)** Remaining % fluorescence signals of Free dye, FDX, mEVs, and FDX@mEVs in GI tract in (A). **(C)** Quantified fluorescence intensity of Free dye, FDX, mEVs, and FDX@mEVs expressed as the radiant efficiency in the GI tract in *ex vivo* images. **(D)** Histological analysis of Free dye, FDX, mEVs, FDX@mEVs, and FcRn in intestinal villi. Scale bar: 50 μm. **(E)** Colocalization analysis between FcRn (green) and mEVs (red) signals. All data presented as mean ± SD, One-way ANOVA with Tukey's *post-hoc* multiple comparisons, ^*^P < 0.05 and ^**^P <0.01, ^***^P < 0.001, ^****^P < 0.0001, *^#^P* < 0.05, ^##^*P* < 0.01, and ^####^*P* < 0.0001. The asterisk (^*^) indicates significance compared to the Free dye group, while the hash (^#^) denotes significance compared to FDX.

The GI tract functions as the primary absorption site for orally administered drugs, a critical consideration in optimizing the effectiveness of oral chemotherapy. To assess *in vivo* intestinal absorption leading to systemic exposure and tumor accumulation, Cy5.5-labeled FDX (**Figure S6**), mEVs, and FDX@mEVs were orally administered to BALB/c mice, followed by near-infrared fluorescence (NIRF) imaging. FDX, administered in an aqueous state as nanoparticles (hydrodynamic size ~220 nm), exhibited rapid *in vivo* clearance within 3 h of administration 22. In contrast, mEVs and FDX@mEVs displayed sustained retention within the GI tract for 24 h post-treatment, as observed in NIRF imaging (**Figure 2A-B**). These results are aligned with our previous report showing the prolonged retention of mEVs in the GI tract, which can be attributed to the distinctive lipid composition characterized by a high phosphatidylethanolamine (PE) ratio in mEVs 24. *Ex vivo* fluorescence images of major organs confirmed a significant abundance of mEVs in the GI tract after 3 h, with Cy5.5 fluorescence signals in the intestine being approximately 4-fold higher in mice treated with mEVs and FDX@mEVs compared to FDX (**Figure 2A-C**). Considering the restricted drug absorption capability of the stomach 25 and the heightened vascularity of the small intestine, characterized by an extensive surface area attributed to tissue villi and enterocyte microvilli 26, our study underscores the benefits of the small intestine, which served as the principal absorption site for the systemic exposure of orally administered drugs. This is achieved through the active targeting of transcytosis receptors, optimizing the process of drug absorption and distribution.

Notably, along with significant FcRn expression in the small intestines compared to the stomach and colon 27 (**Figure S7**), we have also demonstrated a strong colocalization (Pearson's correlation coefficient of 0.7901 for mEVs and 0.8214 for FDX@mEVs) between mEVs (red color) and FcRn (green color) in the small intestine (**Figure 2D-E**). Although a clear mEV signal was also observed in the colon (**Figure S8**), we focused on the intestine due to its higher level of FcRn expression and its role as the primary absorption site for orally administered drugs 28. These results indicate the efficient accumulation and retention of mEVs in the intestine through the interaction with FcRn after oral administration.

### FcRn-mediated intestinal transcytosis and tumor accumulation of mEVs

**Figure 3:**
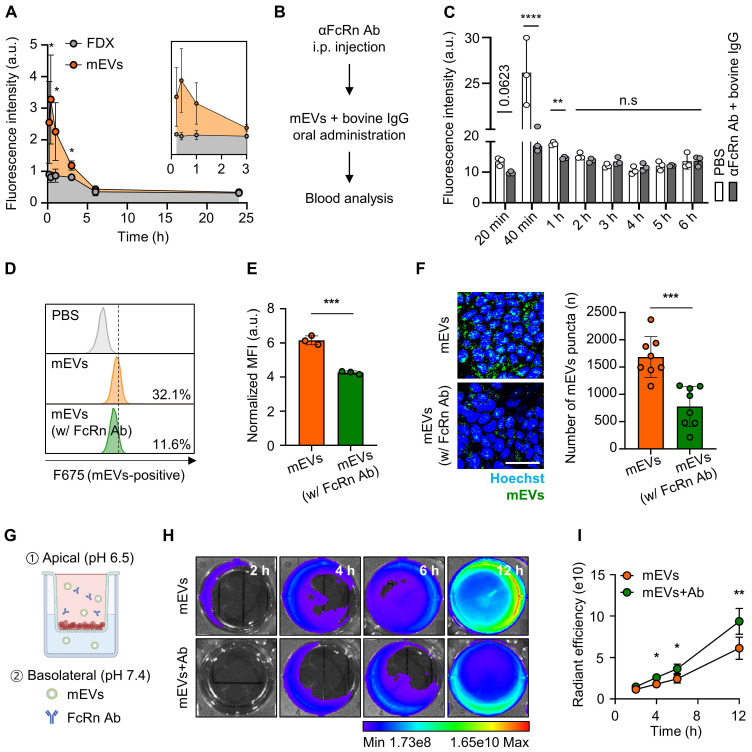
** Validation of transcytosis mechanisms for mEVs intestinal absorption. (A)** Fluorescence-based PK analysis results at various time points (20, 40 min, 1, 3, 6, and 24 h). **(B-C)** Fluorescence signal-based blood analysis at various time points (20 min, 40 min, 1, 2, 3, 4, 5, and 6 h) after oral administration of mEVs. **(D)** Representative histogram of flow cytometry showing a cellular uptake of mEVs in Caco-2 cells after pre-incubation with anti-FcRn antibodies (% in the histogram represents intracellular mEVs-uptaken cell populations). **(E)** Normalized MFI indicating intracellular mEVs-positive cells. **(F)** Representative fluorescence confocal images showing cellular uptake of mEVs in Caco-2 cells with FcRn pre-blocking and quantitative analysis of intracellular mEVs puncta. Cy5.5-labeled mEVs were pseudo-colored in green. **(G)** Schematic demonstrating the Caco-2-based *in vitro* intestinal transcytosis assay. **(H)** The NIRF images to detect Cy5.5-labeled mEVs transported to a basolateral chamber at various time points (2, 4, 6, and 12 h). **(I)** The cumulative quantification of fluorescence signals in (H). Ab indicates anti-FcRn antibodies. All data presented as mean ± SD, Two-tailed t-test, ^**^P < 0.01 and ^***^P < 0.001, and ^****^P < 0.0001.

To assess FcRn binding-mediated active transcytosis of mEVs from the intestines into the systemic circulation, we examined their blood concentration over time after oral administration (**Figure 3A**). Notably, a significant presence of mEVs was observed in the bloodstream for 24 h of treatment; at early time points of 20- or 40-min post-administration, their levels increased by 1.85-fold and 3.59-fold compared to FDX, respectively. Furthermore, to verify whether the mechanism by which orally administered mEVs enter the bloodstream is indeed mediated by FcRn, we conducted an *in vivo* competitive assay using an anti-FcRn antibody (αFcRn Ab) and bovine IgG. 30 min after intraperitoneal injection of the αFcRn Ab, we orally administered bovine IgG and Cy5.5-labeled mEVs simultaneously. We then detected the fluorescence signal of mEVs in the blood at various time points. Blood analysis revealed that 40 min and 1 h after administration, the group administered both αFcRn Ab and bovine IgG showed a reduction in fluorescence intensity of 29.1% and 24.1%, respectively, compared to the group administered with mEVs alone (**Figure 3B-C**). In addition, we confirmed that FDX complexation does not affect the intestinal absorption of mEVs (**Figure S9**). These observations indicate rapid systemic exposure of mEVs through FcRn-mediated transcytosis. Consequently, mEVs demonstrated enhanced pharmacokinetic (PK) properties, with a 1.97-fold higher area under the curve (AUC) and an extended *in vivo* residence time compared to FDX.

To evaluate the substantial FcRn-mediated transcytosis of mEVs, we explored their apical endocytosis, intracellular trafficking, and basolateral exocytosis in cultured conditions. Before proceeding with subsequent experiments, we confirmed the expression of FcRn in the Caco-2 cell line, which we used as a model cell line, as shown by fluorescence imaging results (**Figure S10**). Flow cytometry results indicated a reduction in the cellular uptake of mEVs in CaCo-2 cells pre-blocked with anti-FcRn antibodies (mEVs-positive cells: 11.6%), compared to PBS-treated control cells (32.1%; **Figure 3D-E**). In agreement with the above results, confocal imaging directly visualized the intracellular uptake of mEVs, with fluorescence signals (green color) significantly decreased across the 5 μm thickness of the z-axis within the cells after FcRn pre-blocking (**Figure 3F**). We further investigated whether the mEVs within the Caco-2 cells, existing as a monolayer in the apical chamber of the transwell system, eventually undergo FcRn-mediated transcytosis to the basolateral chamber (**Figure 3G**). This system mimics the intestinal environment in which mEVs bind to FcRn on the apical surface of the epithelium (pH 6.5) and are released due to reduced affinity in the neutral conditions (pH 7.4) of the basolateral side, ultimately entering the bloodstream 29-31. Upon measuring the fluorescence intensities in the basolateral chamber, we observed a decrease in the fluorescence signals of mEVs when the CaCo-2 cells were pre-treated with FcRn antibodies to competitively block the FcRn, compared to control cells (**Figure 3H**). Quantitatively, blocking FcRn significantly reduced the fluorescence signals in the basolateral chamber by 65.4 to 78.6% across all time points (4, 6, and 12 h; **Figure 3I**). This finding strongly suggests that the binding of mEVs to FcRn in the intestinal region can potentially facilitate their transcytosis into the systemic circulation.

**Figure 4:**
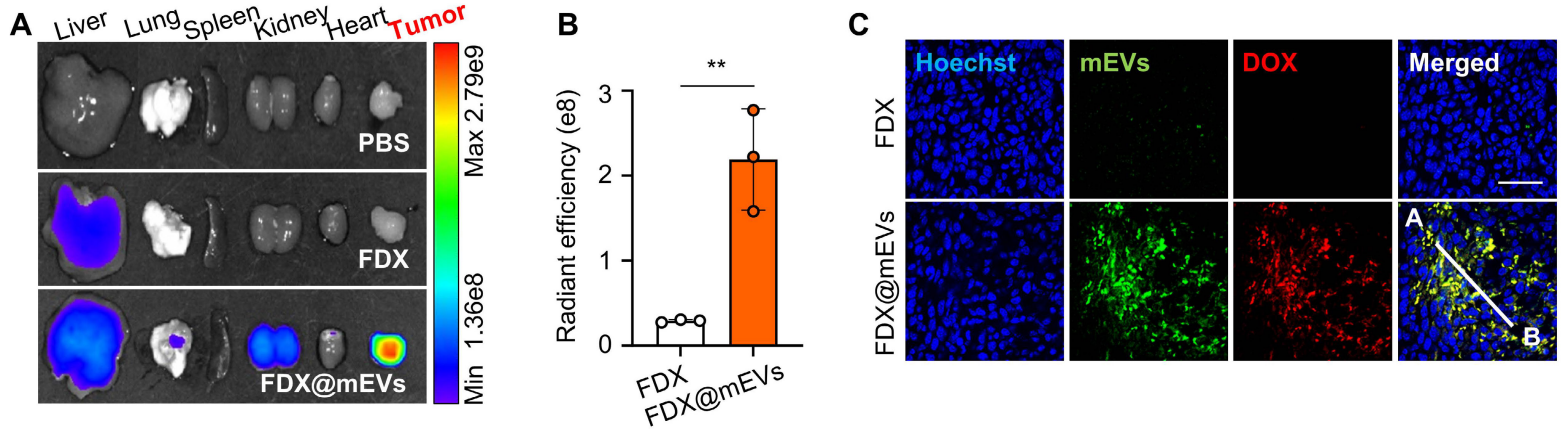
** mEVs-mediated tumor accumulation of FDX after blood circulation. (A)** *Ex vivo* NIRF images of major organs and tumor tissue collected from (**Figure S11**). **(B)** Quantified fluorescence intensity of FDX and FDX@mEVs in tumor tissues in (A). **(C)** Histological analysis of tumor tissues collected from FDX and FDX@mEVs-administered mice. Scale bar: 50 μm. All data presented as mean ± SD, Two-tailed t-test, ^**^P <0.01.

Next, gut-to-tumor oral drug delivery via systemic exposure through FcRn-mediated transcytosis of mEVs was examined in colon tumor models established by subcutaneous inoculation of CT26 cells. *Ex vivo* NIRF images illustrated a substantial accumulation of FDX@mEVs in tumors as they passed through the GI tract, with fluorescence intensities in the tumor tissues 8.87- to 9.54-fold stronger compared to FDX after 24 h of administration (**Figure 4A-B**). The considerable tumor accumulation following systemic exposure to FDX@mEVs can be attributed to the enhanced permeability and retention effect, involving the passive accumulation of nano-sized vesicles through leaky angiogenic blood vessels near tumor tissues 32, 33. Histological analyses demonstrated a robust colocalization (**Figure 4C**) with Pearson's correlation coefficient of 0.9221 between DOX fluorescence and Cy5.5 fluorescence from mEVs in the tumor tissues of mice treated with FDX@mEVs (**Figure S12**). Collectively, these findings signify that the unique properties of mEVs enable efficient delivery of FDX to the tumor site via gut-to-oral drug delivery.

### Antitumor efficacy and safety of FDX@mEVs in colon tumor models

**Figure 5:**
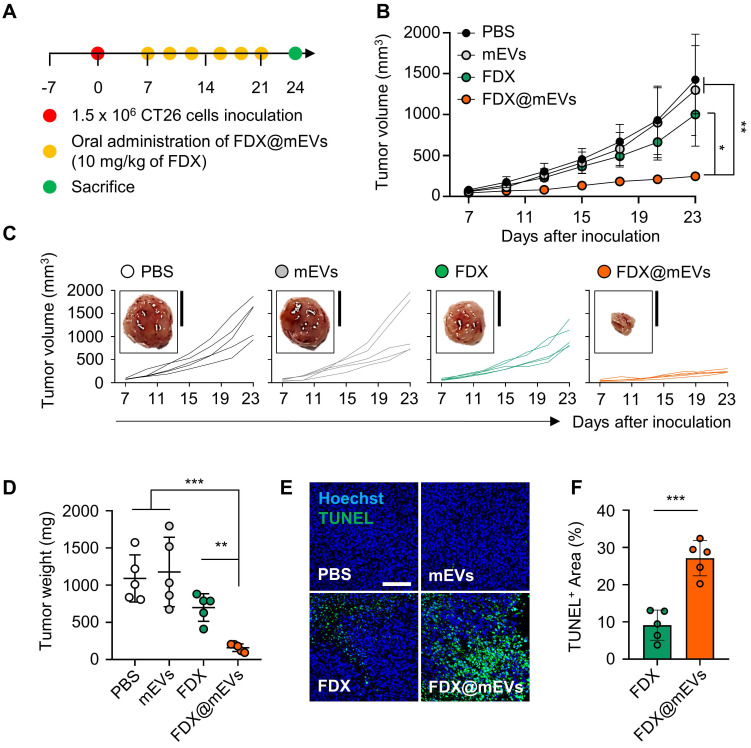
***In vivo* antitumor efficacy of FDX@mEVs in CT26 tumor-bearing mice. (A)** Experimental schedule for *in vivo* therapeutic effects of FDX@mEVs. **(B, C)** Tumor growth curves for 18 days after oral administration of each group (n = 5 for each group; black circle: PBS; grey circle: mEVs; green circle: FDX; orange circle: FDX@mEVs). Black solid boxes show representative tumor tissues dissected from all groups. All tumor tissues collected from all mice are presented in **Figure S13**. Scale bar: 1 cm. **(D)** Tumor weight was measured after three days of the last administration of all groups. **(E, F)** A TUNEL assay for confirming apoptotic cells in tumor tissues and quantified graph demonstrating TUNEL-positive area. Scale bar: 100 μm. All data are presented as mean ± SD. n = 5. One-way ANOVA with Tukey's *post-hoc* multiple comparison and two-tailed t-test, ^*^P < 0.05, ^**^P < 0.01, and ^***^P < 0.001.

The antitumor efficacy of FDX@mEVs was evaluated in colon tumor models, established by subcutaneously inoculating CT26 cells into the left flank of mice. The mice were randomly divided into four groups: (i) PBS; (ii) mEVs; (iii) FDX; and (iv) FDX@mEVs, followed by oral administration of FDX@mEVs at a 10 mg/kg FDX dosage, or an equivalent dose of FDX or mEVs, once every three days (**Figure 5A**). Treatment with FDX@mEVs (237.32 ± 31.08 mm^3^) significantly restrained tumor growth compared to the PBS (1425.51 ± 373.11 mm^3^), mEVs (1298.77 ± 610.9 mm^3^), and FDX (1000.59 ± 229.14 mm^3^) groups (**Figure 5B-C**). In line with reduced tumor growth, we observed a remarkable decrease in tumor weight in the FDX@mEVs group on day 15 after treatment (**Figure 5D**). Furthermore, histological analyses of TUNEL-stained tumor tissues exhibited markedly increased apoptotic cell death throughout the tumor areas in the FDX@mEVs group compared to the other groups (**Figure 5E**). Quantitatively, TUNEL-positive areas in tumor tissues from mice treated with FDX@mEVs significantly increased compared to those treated with FDX (**Figure 5F**).

**Figure 6:**
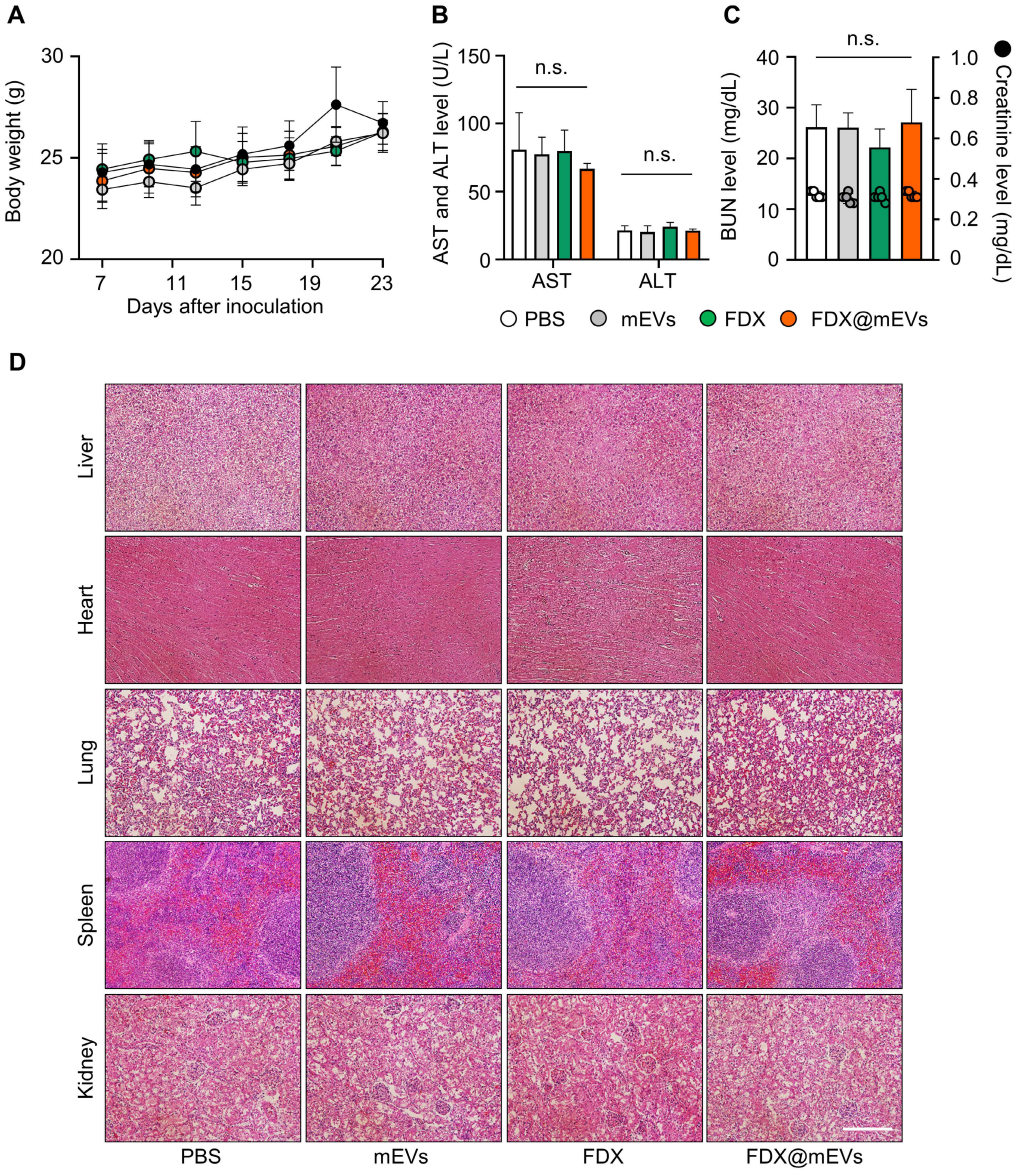
** Systemic toxicity of FDX and FDX@mEVs after oral administration. (A)** Body weight changes in mice during multiple administrations of PBS, mEVs, FDX, and FDX@mEVs (n = 5, black circle: PBS, grey circle: mEVs, green circle: FDX, and orange circle: FDX@mEVs). **(B, C)** Blood analysis for evaluating hematological parameters, such as AST, ALT, BUN, and creatinine. Bar graphs and dots indicate the BUN level and creatinine level, respectively in (C). **(D)** Representative H&E images of major organs (liver, heart, lung, spleen, and kidney). Scale bar: 100 μm. One-way ANOVA with Tukey's post-hoc multiple comparison, n.s.: not significant.

Throughout the repeated oral administration of FDX@mEVs at three-day intervals, no noticeable body weight loss occurred compared to the PBS group (**Figure** 6**A**). Additionally, there were also no significant changes in body weight in the mEVs and FDX groups during treatments. All treatment groups exhibited normal-range hematological parameters related to liver and kidney function, such as alanine transaminase (ALT), aspartate aminotransferase (AST), blood urea nitrogen (BUN), and creatinine, similar to the PBS group (**Figure 6B-C**). Furthermore, histological analyses of major organs stained with hematoxylin and eosin (H&E) did not reveal any structural abnormalities in any of the groups (**Figure 6D**). Taken together, our findings suggest that mEVs could effectively mediate gut-to-tumor oral drug delivery, inhibiting tumor progression while minimizing the risk of side effects on off-target normal tissues.

## Conclusions

Oral administration, a pillar of the pharmaceutical industry, currently encompasses 62% of FDA-approved drugs due to its convenience and high patient acceptance. Despite its prominence, the past five years have witnessed the evaluation of over 3,000 drug candidates for oral chemotherapy in clinical settings, with only SCEMBLIX securing approval for myeloid leukemia treatment. Most compounds have failed due to concerns about efficacy and safety, stemming from their poor pharmacokinetic profiles, low bioavailability, and off-target toxicity. This has led to a concerted effort to explore innovative oral delivery approaches to address these challenges 34. Recent advances highlight the effectiveness of EVs derived from food, such as milk (mEVs), as potent oral drug carriers with exceptional stability under the harsh conditions of the GI tract. A more important discovery is that mEVs can traverse to the bloodstream via interactions with FcRn of the intestinal barrier. This ability makes mEVs a promising tool for enhancing the bioavailability of orally administered drugs, which traditionally struggle with absorption and systemic exposure 35.

In this study, mEVs were complexed with tumor-activated doxorubicin prodrugs for selective treatment. Molecular imaging enabled the direct visualization of mEVs transporting drugs from the intestines to the systemic circulation, subsequently reaching tumor tissues; eventually, mEVs achieved outstanding antitumor efficacy by effectively delivering a prodrug, which struggles with systemic exposure due to the lack of an absorption mechanism in the GI tract upon oral delivery.

When comparing our oral gavage method using mEVs with the intravenous (IV) injection of engineered exosomes reported in other studies 36, several advantages of oral administration become apparent. While IV injection offers rapid action, nearly 100% bioavailability, and precise dosage control, it also comes with drawbacks such as discomfort, pain, and the need for medical resources and trained personnel. Moreover, IV administration can lead to high upfront concentrations of drugs in the bloodstream, potentially causing off-target effects and toxicity. Oral administration, on the other hand, is non-invasive, easier to administer, and generally more acceptable to patients, especially for long-term treatments. This route also allows for a more gradual absorption of the drug, potentially reducing the risk of acute side effects.

Beyond the peptide-conjugated prodrugs used in our research, mEVs have demonstrated the capability to orally deliver a broad range of therapeutic agents, including proteins 37, small molecule drugs 38, and RNA therapeutics 24. This versatility underscores the potential of mEVs as a robust platform for oral drug delivery, capable of addressing the challenges associated with oral administration and expanding the scope of treatable conditions through more effective and patient-friendly therapeutic options.

## Methods

### Isolation of mEVs from the pasteurized low-fat milk

All batches of mEVs were obtained from 400 mL of commercially available low-fat milk that had been pasteurized at 63 ℃. The nutritional information of the milk for mEVs isolation is as follows (**Table 1**):

The isolation process consisted of two consecutive centrifugations at 4 ℃. To remove larger contaminants such as milk fat, the milk was centrifuged at 5,000 x g for 30 min and then 12,000 x g for 1 h using the Avanti J-E High-speed centrifuge equipped with a fixed-angle JA 14 rotor (Beckman Coulter). Following initial centrifugation, the supernatant was further filtered using a 40 μm pore-sized cell strainer (SPL) and then stored at -20 ℃ until further use. For the isolation of mEVs, the centrifuged milk supernatant underwent additional ultracentrifugation at 35,000 x g for 1 h, followed by 70,000 x g for 3 h. After completion of sequential ultracentrifugation, the supernatant was then filtered using 0.8, 0.45, and 0.2 μm pore-sized syringe filters (Sartorius) to eliminate any remaining impurities. The filtered supernatant was then subjected to a final ultracentrifugation step at 100,000 x g for 1 h, using an Optima XE-100 with a fixed-angle 45TI rotor (Beckman Coulter), and the resulting mEVs pellet was resuspended in ice-cold 1X phosphate-buffered saline (PBS, Thermo Fisher). Before using the mEVs, they underwent additional filtration using a 100K MWCO Amicon Ultra-0.5 centrifugal filter (Merck) at 12,000 x g for 10 min. The total amount of mEVs was quantified using a Pierce BCA protein kit (Thermo Fisher) following the manufacturer's instructions.

### Fluorescence labeling of mEVs

For fluorescence analysis-related experiments, mEVs were labeled using a Cy5.5 NHS ester (Bioacts). Briefly, 6.5 mM Cy5.5 NHS ester (stocked in DMSO) was incubated with 1 mg of mEVs O/N at 4 ℃ (total volume: 1 mL with 1% DMSO in PBS; final concentration of Cy5.5 NHS ester: 65 μM). To remove the unlabeled free Cy5.5 NHS ester, labeled mEVs were subjected to 100K MWCO filtration using the 100K Amicon Ultra-0.5 centrifugal filter at 12,000 x g for 10 min.

### Enzyme preparation and incubation of mEVs

All enzymes used in this study, such as α-amylase, pepsin, and lipase (sigma), were prepared following the manufacturer's protocol. Briefly, 1 mg of α-amylase (≥ 1000 units/mg protein) was solved in 1 mL of 20 ℃ ultrapure water. Then, the α-amylase stock was diluted to 1.0 units (1/1000 dilution) for a working solution. The pepsin was stocked in ice-cold 10 mM HCl with ultrapure water. The pepsin stock was subjected to additional dilution (1/100 dilution) using ultrapure water. The lipase was stocked in DW at 1 mg/mL concentration using a bath sonicator. Before use for the experiment, the lipase solution was diluted in DW (1/100 dilution). Subsequently, 200 μg of mEVs were added into 500 μL of each enzyme solution, and incubated for 30 min at 37 ℃. After the incubation, all samples underwent 100K MWCO filtration to remove digestive enzymes and were quantified for the following experiments using BCA assay.

### Immunoblot assay

To confirm EV protein expression after enzyme incubation, 10 μg of mEVs were mixed with D.W and 4X Laemmle sample buffer (Bio-Rad) containing β-mercaptoethanol and heated at 99 ℃ for 10 min. All samples were separated by 12% sodium dodecyl sulfate (SDS)-polyacrylamide gel electrophoresis (PAGE) and transferred to a nitrocellulose membrane using a trans-blot turbo transfer system (Bio-Rad). After completion of gel transfer, the membrane was blocked using 3% bovine serum albumin (BSA) containing Tris-buffered saline with 0.5% Tween 20 (TBST) for 1 h at room temperature (RT). Following the blocking procedure, the membrane was incubated with primary TSG101 (1:1000 in 1% BSA-containing TBST; Abcam, ab83), CD9 (1:1000 in 1% BSA-containing TBST; Novus Biologics, NU500-494), MFG-E8 (1:1000 in 1% BSA-containing TBST; R&D systems, AF2805), GAPDH (1:2000 in 1% BSA-containing TBST; R&D systems, and Hsp70 (1:1000 in 1% BSA-containing TBST; Abcam, ab2787) at 4 ℃ for O/N. Membranes incubated with each primary antibody were washed using TBST and incubated with HRP-conjugated secondary antibody (1:2000 in TBST; GeneTex, GTX213111-01, and GTX231110-01 for anti-mouse and rabbit, respectively, and Abcam, ab6741 for anti-goat) at RT for 1 h. Subsequently, electrochemiluminescent (ECL) substrate solutions (Bio-Rad) were poured onto the membrane for 1 min, and chemiluminescence signals were detected using a ChemiDoc instrument (Thermo Fisher). To collect cell lysate for assessing cathepsin B expression levels in CT26 and NCM460 cells, 3.0 x 10^5^ cells were placed in a 60-mm cell culture dish (SPL). When the confluence of the cells reached approximately 80%, all cells were scraped off using 250 μL of RIPA lysis buffer (Thermo Fisher) and cell scraper (SPL), followed by on-ice incubation for 30 min. After ice incubation, all samples were subjected to centrifugation at 14,000 x g for 20 min. Following centrifugation, the supernatant was collected and quantified using a BCA protein quantification kit. Protein samples obtained from CT26 and NCM460 cells were separated using 12% SDS-PAGE and incubated with cathepsin B antibody (1:1000 in 1% BSA-containing TBST; Santa Cruz Biotechnology, sc-365558) at 4 ℃ for O/N. The following procedure is the same as the protocol mentioned above. To verify IgG expression in mEVs, 3 μg of mEVs were placed onto the nitrocellulose membrane (Total volume: 3 μL/each dot). After 2 h membrane drying at RT, 3% BSA was applied onto the membrane to block non-specific binding. Subsequently, the blocked membrane was incubated with HRP-conjugated anti-bovine IgG antibody (1:2000 in 1% BSA-containing TBST; Invitrogen, A18751) for 1 h at RT. After three times washing with TBST (5 min/each), the membrane was reacted with ECL reagent for 1 min at RT, and chemiluminescence signals were detected using a ChemiDoc.

### Nanoparticle tracking analysis

To determine the size distribution of mEVs incubated in various enzymes and FDX-complexed mEVs, 10 μg of mEVs species were added to 1 mL of filtered PBS. After loading the prepared mEVs species into an NTA instrument (NanoSight LM10, Malvern Panalytical), the measurement was conducted (camera gain: 13, detection threshold: 3) in replicates, totaling 5 repetitions.

### Transmission electron microscopy

mEVs and FDX@mEVs with an equivalent 10 μg of mEVs quantity were placed on the carbon film (total volume: 5 μL in ultrapure water) of 200 mesh copper grids (Electron Microscopy Sciences) for 5 min at RT. To remove excess sample solution, sample-loaded copper grids were absorbed by filter papers. Afterward, all samples were incubated with 2% uranyl acetate solution for 30 s for negative staining, followed by sample drying O/N at RT. TEM imaging was conducted using a Tecnai F20 G2 transmission electron microscope (TEI).

### Preparation of FDX@mEVs

400 μg of FDX (stocked at 10 μg/μL in DMSO) and 400 μg of mEVs were incubated (total volume: 400 μL with PBS; final DMSO %: 10% (*v/v*)) for 1 h at 37 ℃ with shaking (1000 rpm) using a programmable thermomixer (KBT). After 1 h incubation, FDX-complexed mEVs were subjected to 100K MWCO filtration to remove uncomplexed free FDX from the FDX@mEVs complex. To calculate complex efficiency, a standard curve was established by measuring serially diluted FDX (ranging from 400 μg/200 μL to 6.25 μg/200 μL) absorbance at 485 nm. Subsequently, the uncomplexed FDX was measured, and the quantity of FDX was calculated based on the standard equation. The complex efficiency and capacity were determined using the following formula:



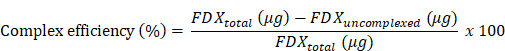





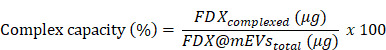



### Analysis of fluorescence spectra of FDX@mEVs

To confirm the complexation between FDX and mEVs, fluorescence was measured after fractionation using a PD-10 column (Cytiva). Firstly, the column storage solution was poured off and the column was filled up with 1X PBS for column equilibration. After 4 times 1X PBS exchanges, 2 mg of mEVs, FDX, and FDX@mEVs (equivalent to 1 mg/mL of FDX concentration; total volume: 2.5 mL) were loaded into the column. Subsequently, 3.5 mL of 1X PBS was added to the column for the elution of samples. The five droplets of the sample were considered as one fraction. After sample collection fluorescence spectra of all samples at the fraction were analyzed using F-7000 Fluorescence Spectrophotometer (HITACHI).

### Super-resolution microscope imaging of FDX@mEVs

50 μg/10 μL of FDX@mEVs were mixed with 10 μL of Fluoromount-G mounting medium and then placed on slide glass. After sample covering using cover glass, SRM imaging was conducted using Elyra7 (ZEISS). Imaging processing was performed using ZEN lite software.

### Stability evaluation of FDX@mEVs

100 μg of FDX@mEVs (based on the number of mEVs) complex was added to 1 mL DW-filled transparent cuvette to evaluate hydrodynamic size changes of the complex at various time points (0, 1, 2, 4, 8, and 24 h). To confirm the serum stability of the FDX@mEVs complex, 500 μg of FDX@mEVs (based on the number of mEVs) was added to 500 μL of mouse serum. Mouse serum was prepared according to the protocol provided by Thermo Fisher. Complex-added mouse serums were incubated at 37 ℃ for 1, 2, 4, 8, and 24 h using a programmable thermomixer. At each time point, FDX separated from mEVs was eliminated through a 100K MWCO filtration, and the FDX present in the complex was measured using a UV-vis spectrophotometer.

### *Ex vivo* digestion assay of mEVs and FDX@mEVs

The *ex vivo* digestive system was prepared using premade artificial saliva containing α-amylase and lysozyme (pH 6.75, TB0928, TMALAB), simulated gastric fluid containing pepsin (pH 1.5, TB1210, TMALAB), artificial bile juice containing bile salt (pH 8.2, TB1220, TMALAB), in-house pancreatic juice containing pancreatin (P3292, Sigma) (pH 8.1, formulated the same composition as in previous study 39). 200 μg of mEVs and FDX@mEVs were incubated with 150 μL of artificial saliva at 37℃ for 5 min, followed by incubation with 300 μL of gastric juice at 37℃ for 120 min. Then, 300 μL of pancreatic juice and 150 μL of bile juice were added to all samples and incubated at 37℃ for 60 min. After the last incubation, all samples underwent centrifugation using a 100K MWCO filter to remove digestive juices and were resuspended in PBS. The size distribution and concentration were determined using NTA.

### Cell culture

NCM460 (normal human colon epithelial cell line) were cultured in Dulbecco's modified Eagle's medium (DMEM, Hyclone) supplemented with 10% fetal bovine serum (FBS, Gibco) and 1% antibiotics-antimycotic (Gibco). CT26 (murine colorectal carcinoma cell line) was maintained in RPMI1640 medium (Welgene) with 10% FBS and 1% antibiotics-antimycotic solution. Caco-2 (human colorectal carcinoma cell line) was cultured in Minimum Essential Medium (MEM, Welgene) with 10% FBS and 1% antibiotics-antimycotic. All cells were incubated at 37 ℃ with 5% CO_2_ level.

### Cellular uptake of FDX@mEVs

2.0 x 10^5^ cells of NCM460 and CT26 cells were placed in a 35-mm glass bottom confocal dish (SPL). After 24 h, DOX, FDX, and FDX@mEVs were treated with an equivalent dose of 1 μM. After 6 h incubation, all cells were washed three times with pre-warmed Dulbecco's phosphate-buffered saline (DPBS) for 5 min, and fixed with 4% PFA for 10 min at RT. The fixed cells were washed with DPBS, and incubated with Hoechst 33342 solution (Invitrogen) for 7 min. After staining, all samples were subjected to DPBS washing, and filled with 2 mL of DPBS to prevent the sample drying. The prepared samples were imaged using a confocal microscope (Leica TCS SP5, Leica).

### Cathepsin B inhibition assay

2.0 x 10^5^ CT26 cells were seeded in a 35-mm glass bottom confocal dish. Once stabilized, the cells were treated with PBS and 50 μM CA-074 (cathepsin B inhibitor). After 6 h of cathepsin B inhibition, FDX@mEVs (adjusted to an FDX concentration of 1 μM) were treated in the cells. Confocal imaging was conducted 6 h after the treatment of FDX@mEVs.

### Flow cytometry analysis

To quantify internalized mEVs in Caco-2 cells, 5.0 x 10^5^ cells were seeded into a microcentrifuge tube filled with 500 μL of PBS. Prior to treating mEVs to cells, 10 μg of FcRn antibody (Santa Cruz Biotechnology, sc-166413) were treated to cells for 1 h at 37 ℃. After the pre-blocking procedure, all groups were incubated with 100 μg of Cy5.5 675-labeled mEVs for 30 min at 37 ℃. Subsequently, all cells were subjected to centrifugation at 1,200 x g for 5 min to wash excess mEVs. After pellet resuspension using PBS, Cy5.5 fluorescence signals were detected using a CytoFLEX flow cytometer (Beckman Coulter).

### *In vitro* transcytosis assays

Transepithelial transport analysis of mEVs was conducted by the methodology outlined in the previous study 27. Briefly, 3.5 x 10^5^ Caco-2 cells were seeded in the apical chamber of the co-culture system (SPLInsert™, SPL). Upon confirming the development of the Caco-2 cell monolayer by measuring transepithelial electrical resistance (TEER, 254.1 Ω ± 31.2) using EVOM Manual instrument (World Precision Instruments) equipped with STX4 electrode, the medium was changed to Hank's balanced salt solution (HBSS) (pH 6.5) in the apical chamber and HBSS (pH 7.4) in the basolateral chamber. 50 μg/mL of Cy5.5-labeled mEVs were treated, and transportation of mEVs to the basolateral chamber was subsequently monitored at various time points (2, 4, 6, and 12 h) using IVIS® Lumina imaging instrument (PerkinElmer). For FcRn pre-blocking, 10 μg of FcRn antibody was added to the apical chamber 1 h before mEVs treatment. Quantitative analysis was carried out using Lumina software.

### Biodistribution of mEVs

All animal experiments were performed following the International Guide for the Care and Use of Laboratory Animals and were approved by the Korea Institute of Science and Technology. 7-week-old BALB/c mice (n = 3 per each time point) were used to monitor the *in vivo* biodistribution of mEVs. To comparatively evaluate the biodistribution of mEVs and FDX, 1 wt% of Cy5.5-conjugated FRRG-DOX was co-assembled with unmodified FRRG-DOX, and then the resulting Cy5.5-FRRG-DOX or Cy5.5-labeled mEVs with an equivalent Cy5.5 fluorescence intensity were administered to the mice **(Figure S4)**. Fluorescence signals in the GI tract were monitored by NIRF imaging. Following the assessment of GI tract distribution, all mice were anesthetized using 2.5% isoflurane gas and dissected to extract their major organs (liver, lung, spleen, heart, and kidney) along with the GI tract, and blood for PK analysis. PK analysis was performed by measuring the fluorescence signal of FDX and mEVs present in the blood at each time point (20 min, 40 min, 1 h, 3 h, 6 h, and 24 h).

### Immunohistochemical analysis of small intestine

Following *ex vivo* fluorescence imaging, the small intestine regions were skillfully rolled using the Swiss-roll technique. The Swiss-rolled intestine tissues underwent a process of dehydration and embedding in paraffin. To elaborate, the tissues were sequentially immersed in 30%, 50%, 70%, 80%, 90%, 95%, and 100% ethanol for 45 min each. Subsequently, all tissues were immersed in xylene three times for 30 min each. Following dehydration, the tissue samples were embedded in paraffin at 58 ℃ for O/N. The embedded tissues were sectioned to a thickness of 6 μm and floated in a 56 ℃ floating bath. The sections were then mounted onto adhesive microscope slides (MARIENFELD). Before conducting immunostaining, the antigen retrieval process was conducted using 100X antigen retrieval buffer (Abcam) for 10 min under boiling using the microwave. After cooling down, all sections were then incubated with FcRn antibodies (1:250, Santa Cruz Biotechnology) at 4 ℃ O/N. Following this, Alexa Fluor 488-conjugated secondary antibodies were applied (1:500, Invitrogen) for 1 h at RT. After three PBS washes, all slides underwent counterstaining for 7 min using Hoechst 33342 (1:2000; Invitrogen). Subsequently, all slides were covered with cover glass using Fluoromount-G mounting medium (Invitrogen). Fluorescence imaging was carried out using confocal microscopy.

### Tumor accumulation of FDX and FDX@mEVs

To verify tumor accumulation of FDX and FDX@mEVs after systemic circulation, 1.5 x 10^6^ CT26 cells were inoculated to the left flank of 7-week-old immunodeficient BALB/c nude mice (CAnN.Cg-Foxn1nu/Crl, n = 3 per each group). When the implanted tumor size reached approximately 180 - 200 mm^3^, 50 mg/kg of FDX@mEVs and FDX (with equivalent Cy5.5 fluorescence intensity) were orally administered. 24 h after administration, all mice underwent *in vivo* NIRF imaging and were sacrificed to collect major organs with tumor tissue. *Ex vivo* imaging was conducted using the IVIS® Lumina instrument. Fluorescence quantification was performed using Lumina software.

### Histological analysis of tumor tissues

After completion of *in vivo* and ex vivo tumor imaging, all collected tumor tissues were embedded in the Tissue-Tek O.C.T compound and stored at -80 ℃. Embedded tissues were sectioned at 10 μm thickness using a CM1950 cryostat (Leica). All sections were mounted on adhesive slide glass, and subjected to counterstaining using Hoechst 33342 (1:2000). Following the Hoechst staining, all samples were covered with a coverglass. Fluorescence imaging of FDX and FDX@mEVs was conducted using confocal microscopy. Correlation analysis was performed using Las X software.

### Antitumor effects of FDX@mEVs

To evaluate the therapeutic efficacy of FDX@mEVs, all controls and FDX@mEVs (PBS, mEVs, FDX, and FDX@mEVs; an equivalent dose of 10 mg/kg of FDX dispersed in 100 μL of PBS) were orally administered to CT26 tumor-bearing mice when the average tumor size reached approximately 50 - 70 mm^3^. The CT26 tumor-bearing mice were modeled in the same manner as the previous method by inoculating 1.5 x 10^6^ cells into the left flank of 7-week-old BALB/c mice (BALB/cAnNCrlOri). The six-repeated administration was conducted every three days, and tumor sizes and body weights were measured every two days. All subjects were sacrificed three days after the last administration, and blood, major organs, and tumor tissues were collected for the following analysis.

### TUNEL assay

Dissected tumor tissues were fixed with 4% PFA for 24 h. Following fixation, all fixed tissues were subjected to dehydration and paraffin embedding. Praffine-embedded tumor tissues were sectioned at 6 μm thickness, and a TUNEL assay (Promega) was conducted to confirm cancer cell apoptosis according to the manufacturer's instruction. The TUNEL-positive area was confirmed by using confocal microscopy, and their intensity was analyzed using Fiji Image J software.

### Systemic toxicity of FDX@mEVs

Systemic toxicity of FDX@mEVs was assessed by analyzing hematological parameters and histological assay of major organs. To assess liver damage by FDX@mEVs, AST, and ALT levels were analyzed. In addition, BUN and creatinine levels were also evaluated to confirm renal toxicity. Subsequently, all major organs were used for H&E staining to verify the histological damage of FDX@mEVs. H&E staining was performed according to the same protocol used in our previous study. Briefly, all sections were incubated in Hematoxylin solution for 5 min at RT. All incubated tissue sections were washed using tap water for 3 min. Then, all samples underwent a bluing procedure for 15 s. Following tap water washing, all samples were incubated in Eosin Y solution for 3 min. After incubation with Eosin Y solution, section-mounted slides were rinsed using absolute alcohol.

### Statistical analysis

A statistical analysis was performed using Prism 10.0 (GraphPad). Statistical significance was determined using a two-tailed t-test (for comparing two groups) and one-way analysis of variance (ANOVA) with Tukey's post-hoc test (for comparing two more groups). A P-value less than 0.05 was considered statistically significant.

### Data availability

All relevant data are available with the article and its Supporting Information files or available from the corresponding authors upon reasonable request.

## Supplementary Material

Supplementary figures.

## Figures and Tables

**Table 1:** The nutrients of commercial milk for mEVs isolation

Nutrient	Amount	Daily Value (%)
Sodium	50 mg	3%
Carbohydrates	5 g	2%
Sugars	5 g	5%
Fat	1.5 g	3%
Trans fat	0 g	-
Saturated fat	1 g	7%
Cholesterol	10 mg	3%
Protein	3 g	5%
Calcium	100 mg	14%

## Abbreviations

ALT: alanine transaminase
AST: aspartate aminotransferase
AUC: area under the curve
BSA: bovine serum albumin
BUN: blood urea nitrogen
DMSO: dimethyl sulfoxide
DOX: doxorubicin
FcRn: neonatal FC receptor
FDX: FRRG-DOX
GI: gastrointestinal
H&E: hematoxylin and eosin
Hsp70: heat shock protein 70
IV: intravenous
mEVs: milk-derived extracellular vesicles
MWCO: molecular weight cut-off
NTA: nanoparticle tracking analysis
PK: pharmacokinetic
SDS: sodium dodecyl sulfate
SRM: super-resolution microscopy
TSG101: tumor susceptibility gene 101
αFcRn Ab: anti-FcRn antibody
