# Supplementary material for: Milk-derived extracellular vesicles enable gut-to-tumor oral delivery of tumor-activated doxorubicin prodrugs
Source: Theranostics. 2024 Aug 26;14(14):5413–28. doi: 10.7150/thno.97269 (PMC11413795; doi:10.7150/thno.97269)
Supplement: Supplementary file 1 — Supplementary figures. [file thnov14p5413s1.pdf]

Supplementary Information for

Milk-derived extracellular vesicles enable gut-to-tumor oral delivery of tumor-activated doxorubicin prodrugs

Hochung Jang[1,2], Jiwoong Choi[1], Daeho Park[1,3], Geonhee Han[1,5], Eun Hye Kim[1,3], Kwangmeyung Kim[4], Sun Hwa Kim[1,5], Man Kyu Shim[1,] and Yoosoo Yang[1,2,*]*

[1]Medicinal Materials Research Center, Biomedical Research Division, Korea Institute of Science and Technology (KIST), Seoul, 02792, Republic of Korea.

[2]Division of Bio-Medical Science and Technology, KIST School, Korea University of Science and Technology, Seoul 02792, Republic of Korea.

[3]Department of Life Sciences, Korea University, Seoul, 02841, Republic of Korea

[4]College of Pharmacy, Graduate School of Pharmaceutical Sciences, Ewha Womans University, Seoul 03760, Republic of Korea.

[5]KU-KIST Graduate School of Converging Science and Technology, Korea University, Seoul, 02841, Republic of Korea.

*Correspondence and requests for materials should be addressed to **Yoosoo Yang** (E-mail: ysyang@kist.re.kr) and **Man Kyu Shim** (E-mail: mks@kist.re.kr).

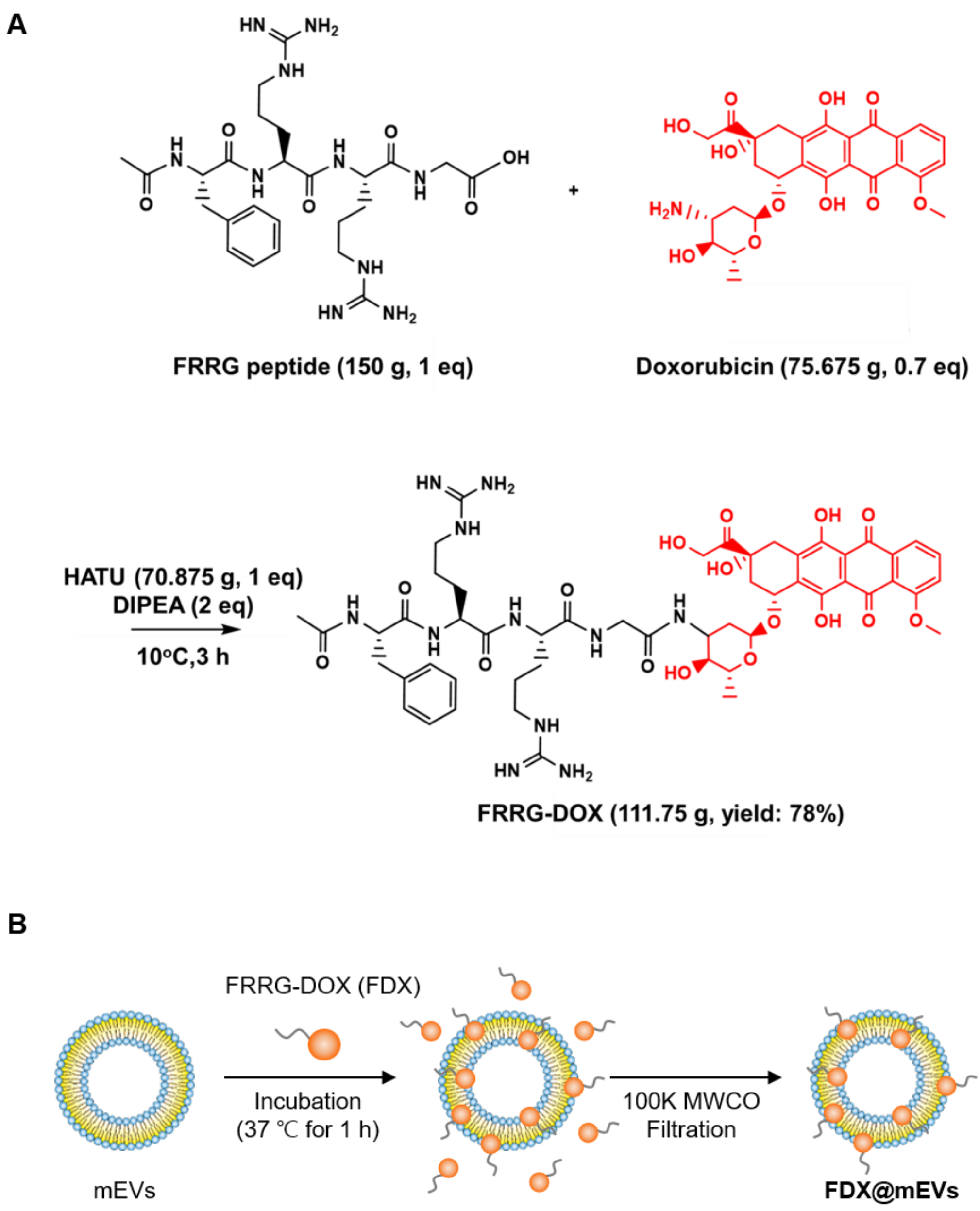

Figure S1. (A) Synthetic route for preparing the tumor-activated doxorubicin prodrug, FDX. **(B)** Schematic representation of the methodology used to prepare the mEVs complexed with FDX.

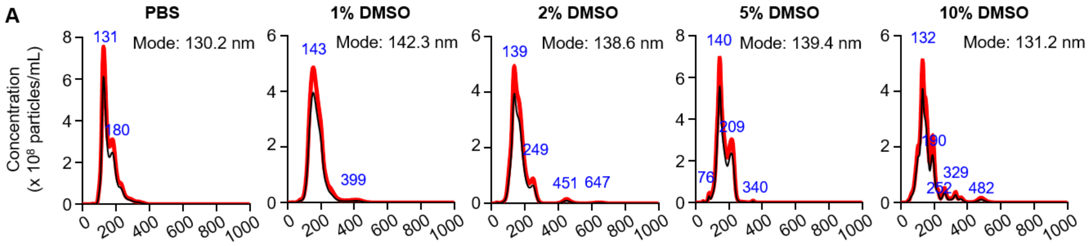

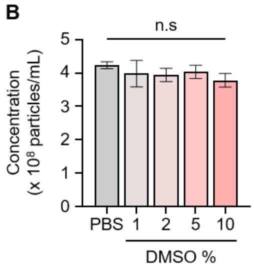

C

1 h incubation at 37 ℃

Group	Concentration (x 10^8 particles/mL)	SD	N
PBS	4.23 x 10^8	1.05 x 10^7	5
1% DMSO	3.98 x 10^8	4.00 x 10^7	5
2% DMSO	3.94 x 10^8	1.98 x 10^7	5
5% DMSO	4.06 x 10^8	1.98 x 10^7	5
10% DMSO	3.78 x 10^8	2.05 x 10^7	5

Figure S2. (A-C) Size distribution and concentration of mEVs after incubation in various DMSO % (*v/v*)

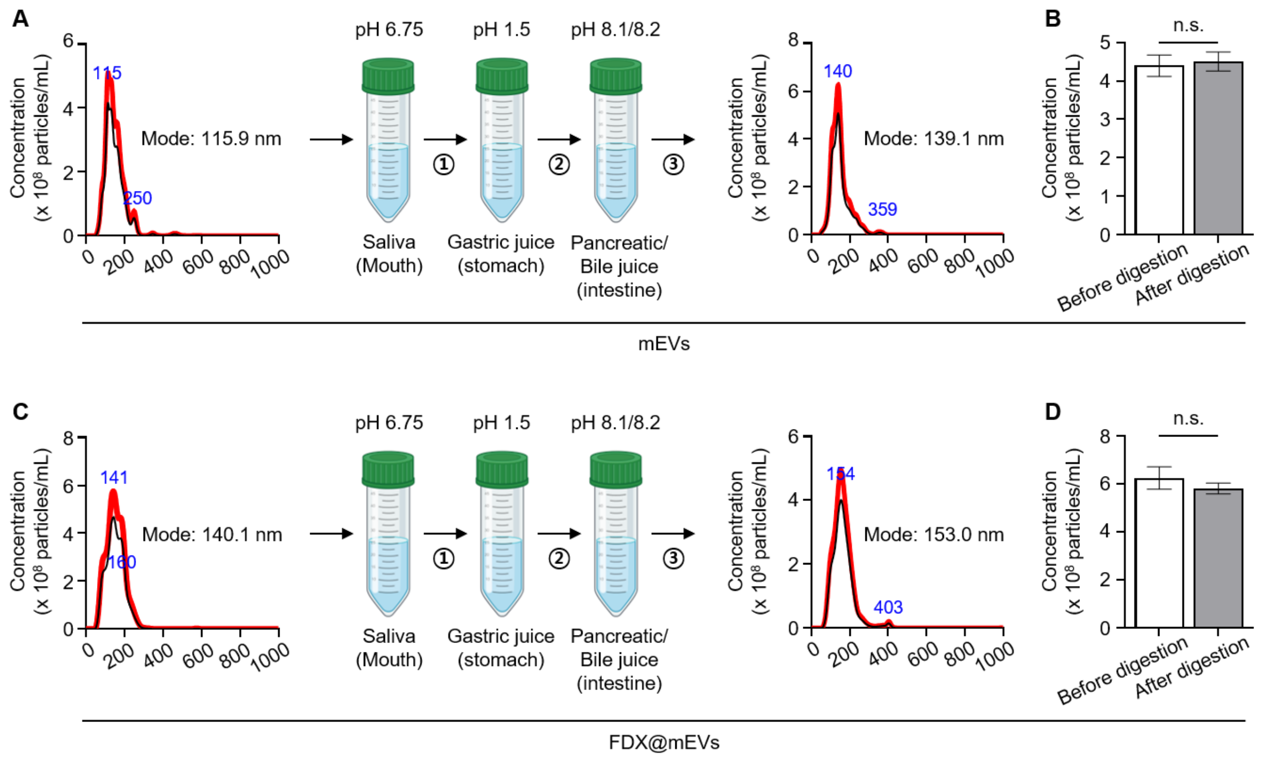

Figure S3. (A-B) Size distribution and concentration of mEVs after incubation in *ex vivo* digestive system. **(C-D)** Size distribution and concentration of FDX@mEVs after incubation in *ex vivo* digestive system. 1: 37℃, 5 min; 2: 37℃, 120 min; 3: 37℃, 60 min.

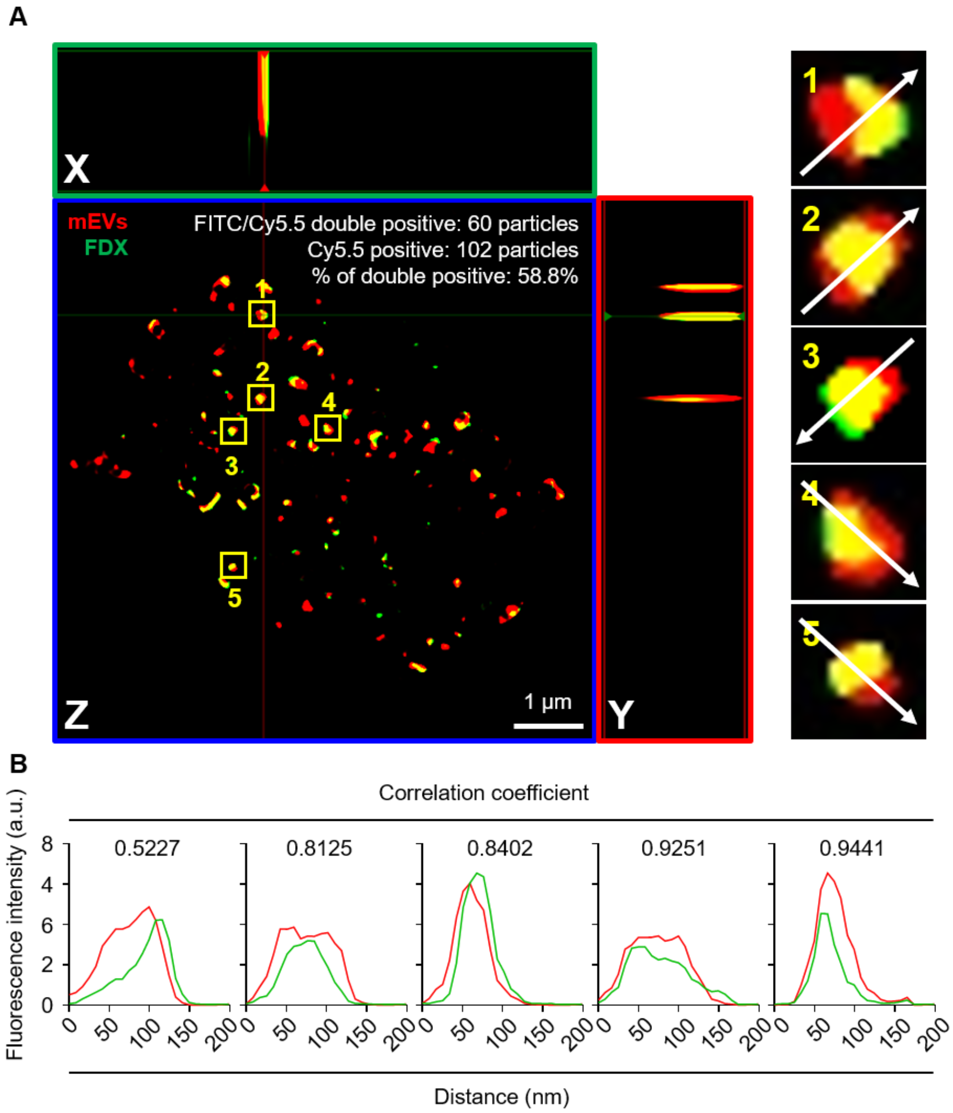

Figure S4. (A) Super-resolution microscopy image of FDX@mEVs. **(B)** Correlation coefficient of representative FDX@mEVs.

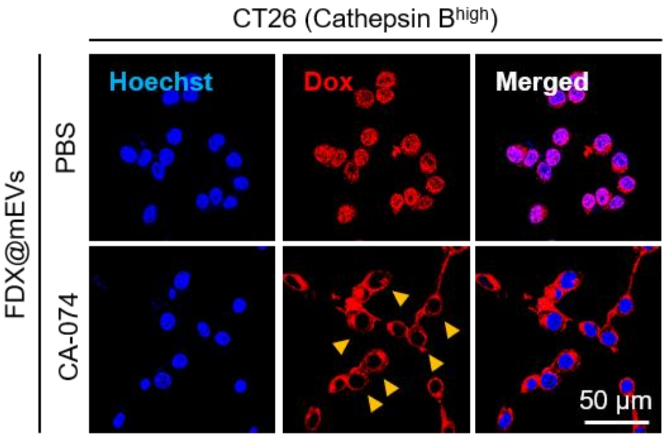

Figure S5. The nuclear translocation behavior of FDX depending on cathepsin B inhibition

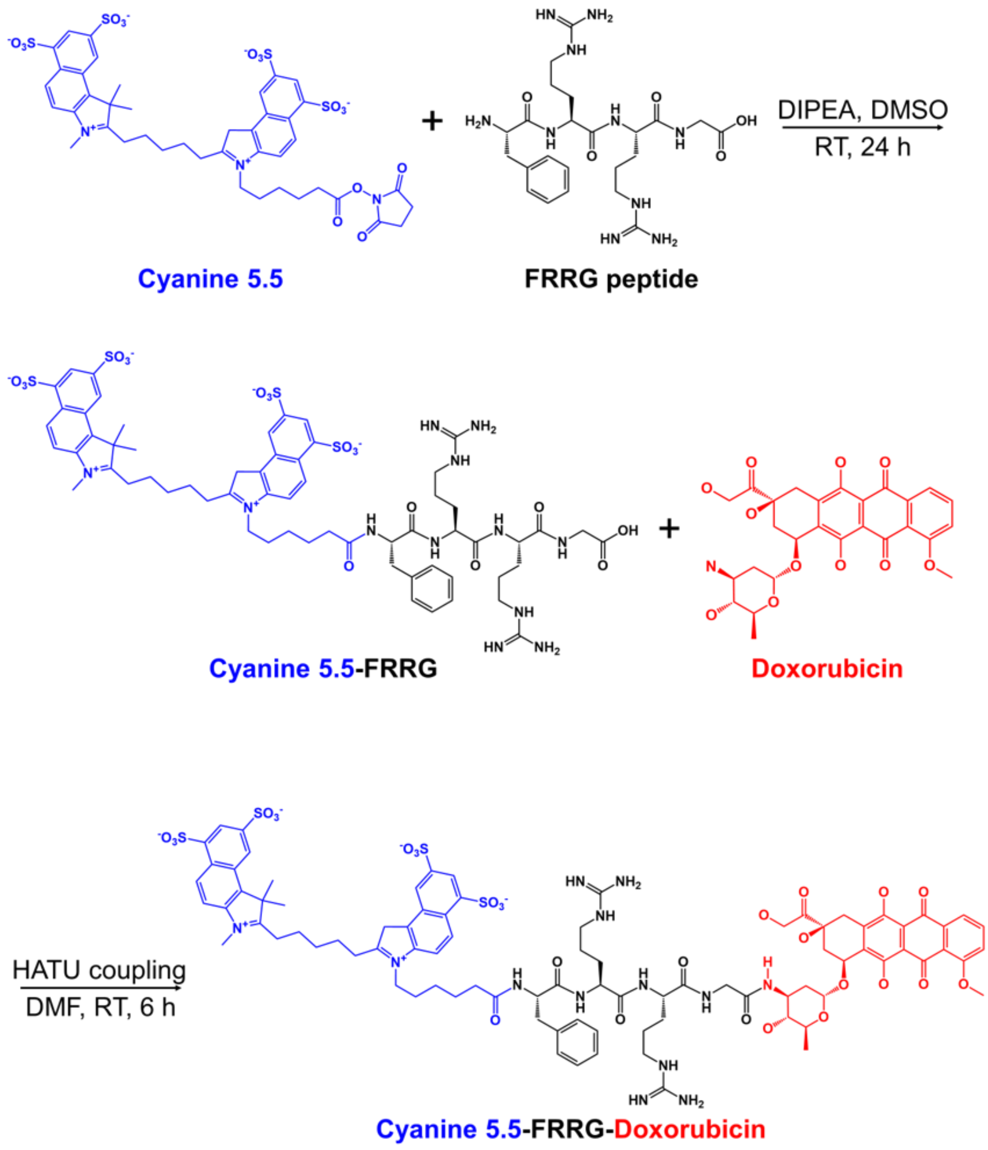

Figure S6. Synthetic route for preparing the Cy5.5-labeled FDX.

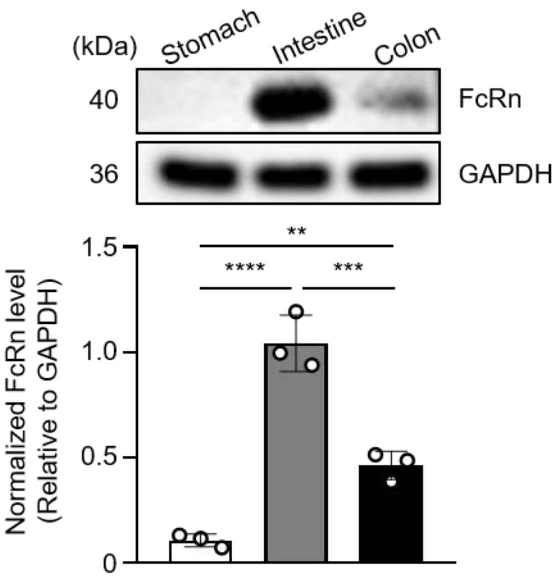

Figure S7. FcRn expression levels in mouse stomach, intestine, and colon

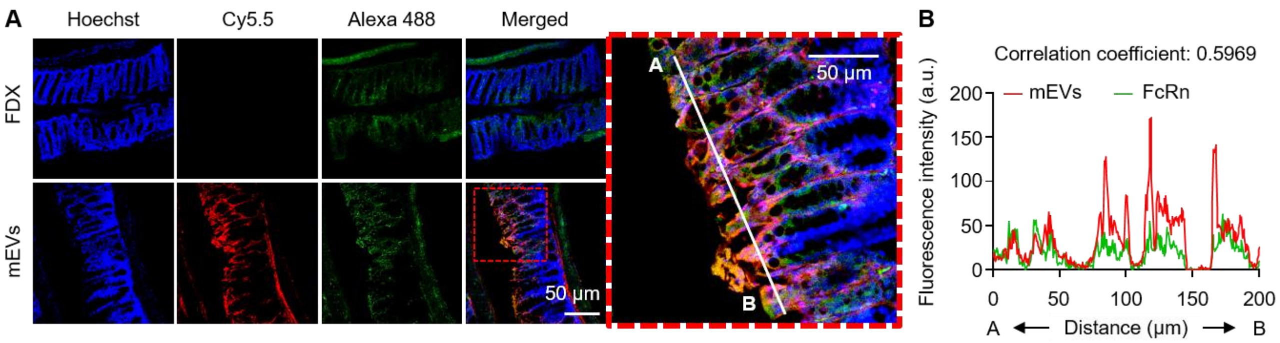

Figure S8. (A-B) The histological analysis of FDX, mEVs, and FcRn in the colon region.

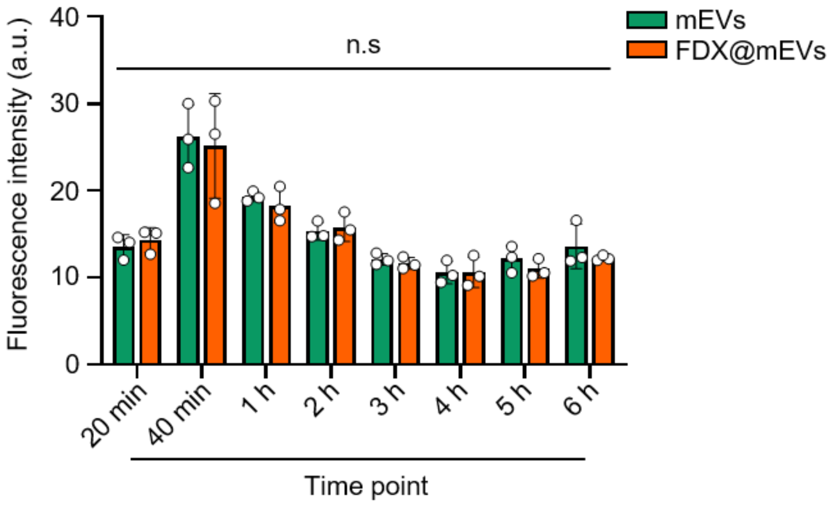

Figure S9. Blood analysis after oral administration of mEVs and FDX@mEVs

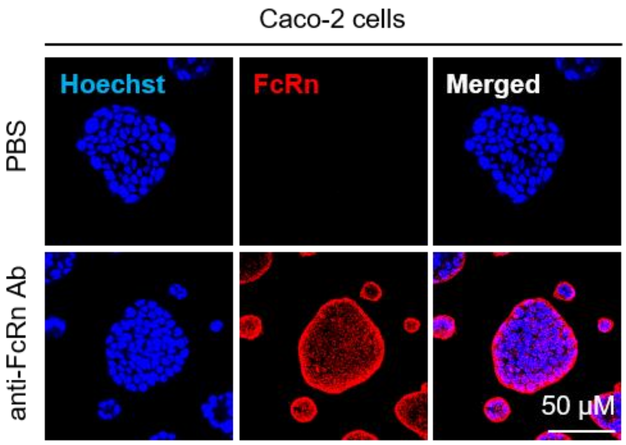

Figure S10. FcRn expression in Caco-2 cell line

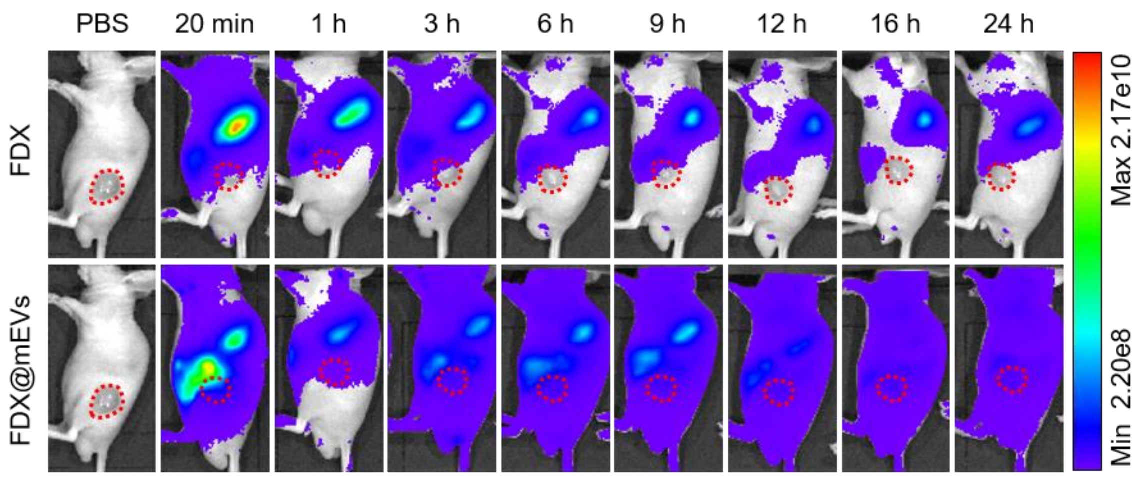

Figure S11. In vivo NIRF whole-body imaging after oral administration of FDX and FDX@mEVs in CT26 tumor-bearing mice at various time points (20 min, 1 h, 3 h, 6 h, 9 h, 12 h, 16 h, 24 h).

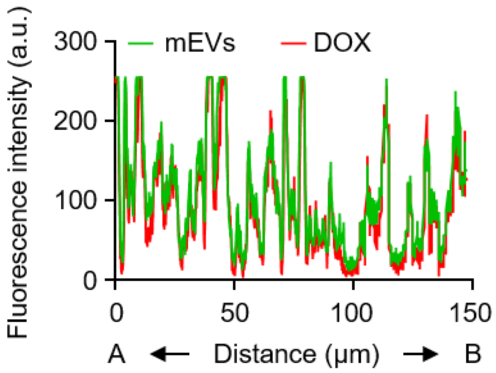

Figure S12. Correlation analysis between mEVs (green) and DOX (red) fluorescence signals in Figure 4C.

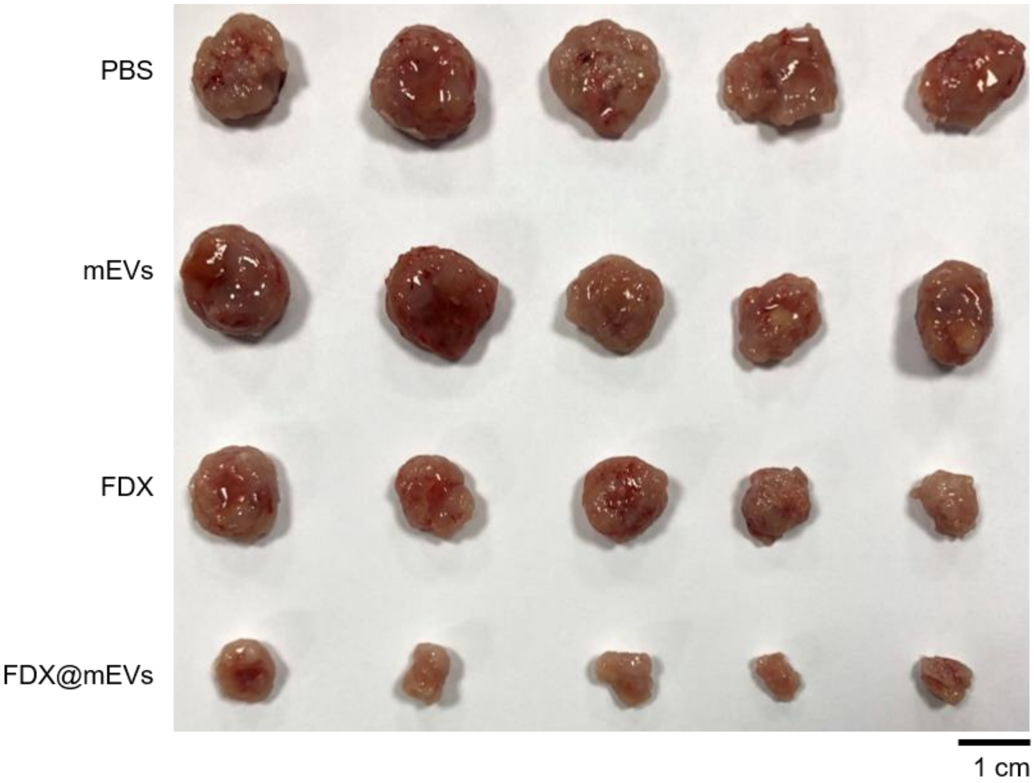

Figure S13. Photo of tumor tissues dissected from all tumor-bearing mice. Scale bar: 1cm.
